# Supplementary material for: Reversibly growing crosslinked polymers with programmable sizes and properties
Source: Nat Commun. 2023 Jun 6;14:3302. doi: 10.1038/s41467-023-38768-z (PMC10244334; doi:10.1038/s41467-023-38768-z)
Supplement: Supplementary file 1 — Supplementary information [file 41467_2023_38768_MOESM1_ESM.pdf]

Supplementary information for

Reversibly growing crosslinked polymers with programmable sizes and properties

Xiaozhuang Zhou[1,2‡], *Yijun Zheng*[3‡], *Haohui Zhang*[4], *Li Yang*[1], *Yubo Cui*[1], *Baiju P. Krishnan*[2], *Shihua Dong*[1], *Michael Aizenberg*[5], *Xinhong Xiong*[1], *Yuhang Hu*[4,6], *Joanna Aizenberg*[5,7*], *Jiaxi Cui*[1,2,5*]

[1] Institute of Fundamental and Frontier Sciences, University of Electronic Science and Technology of China, Chengdu, Sichuan 610054, China

[2] INM - Leibniz Institute for New Materials, Campus D2 2, 66123, Saarbrücken, Germany

[3] School of Physical Science and Technology, ShanghaiTech University, Shanghai 201210, China

[4] The George W. Woodruff School of Mechanical Engineering, Georgia Institute of Technology, Atlanta, Georgia 30332, United States

[5] John A. Paulson School of Engineering and Applied Sciences, Harvard University, Cambridge, Massachusetts 02138, USA

[6] The School of Chemical and Biomolecular Engineering, Georgia Institute of Technology, Atlanta, Georgia 30332, United States

[7] Department of Chemistry and Chemical Biology, Harvard University, Cambridge, Massachusetts 02138, USA

‡ These authors contributed equally: Xiaozhuang Zhou, Yijun Zheng.

*To whom correspondence should be addressed. Email: Jiaxi.Cui@uestc.edu.cn (J.C.); jaiz@seas.harvard.edu (J.A.)

Supplementary Note 1: acid-catalyzed ring-opening polymerization and equilibration of siloxane

1.1 *Acid-activated polymerization and related reactions*

The ring-opening polymerization of D_4 (one of the most common siloxanes) is accompanied by various processes of depolymerization, repolymerization, and chain transfer and the nature of the active center in polymerization is still obscure[1-4]. Supplementary Figure 1 shows the main reactions occurring in the polymerization with trifluoromethanesulfonic acid as a catalyst and the complex active centers are represented by "*". The *initiation* of the polymerization starts from the electrophilic attack of the proton of trifluoromethanesulfonic acid on a siloxane ring oxygen and the opening of the ring, resulting in an active center that can attack cyclic monomers to propagate (*propagation*). A *backbiting* reaction would lead to the reformation of the cyclic monomers, accompanied by the decrease of the molecular weight of the polymer chains (depolymerization). In the presence of acidic species, two polymer chains can undergo chain-exchange reactions (*rearrangement*). The system is living and maintains a dynamic equilibrium state.

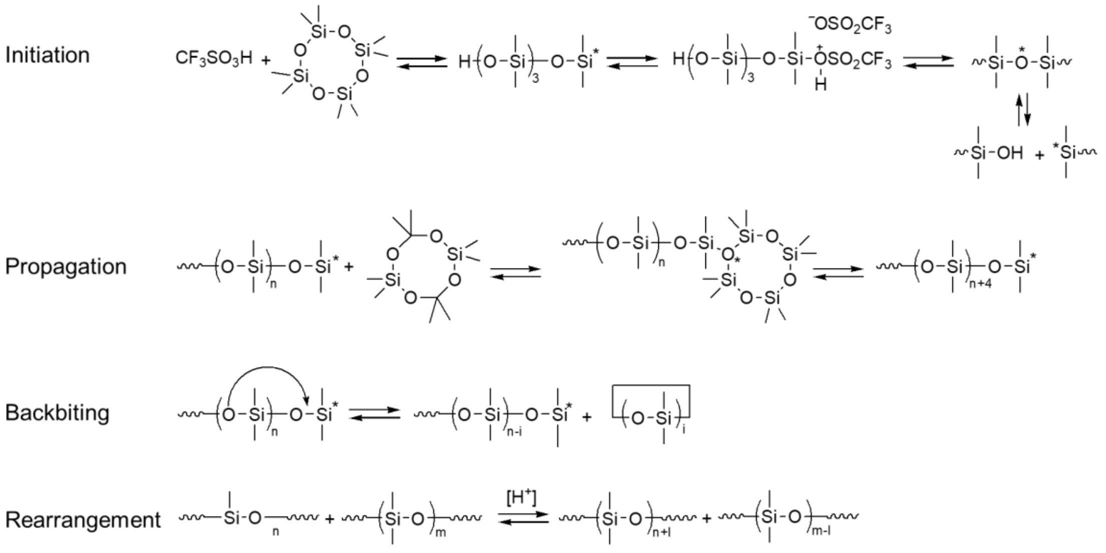

Supplementary Figure 1. Acid-catalyzed ring-opening polymerization of D_4 and related reactions.

1.2 *Transfer of active living species*

Supplementary Figure 2 shows the transfer mechanism of active living species (redistribution of the active living species). In the acid-catalyzed siloxane formation, the transfer of the active living species involves the dissociation and re-bonding of the Si-O bond[1,4]. The transfer of active species can occur at room temperature (rt) and therefore, homogeneous distribution of active species is readily established at rt. On the other hand, the transfer rate of active species is comparable to the rate of acid-catalyzed polymerization[4].

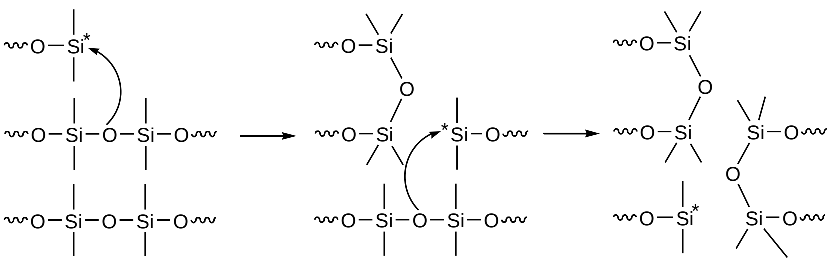

Supplementary Figure 2. Mechanisms of transfer of the active living species in the siloxane polymer networks.

Supplementary Note 2: synthesis

2.1 *1,1,1-tri(2-heptamethylcyclotetrasiloxane-yl-ethyl)-methylsilane (triD$_4$)*

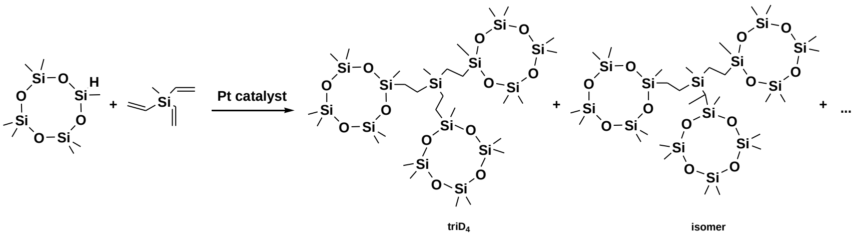

Supplementary Figure 3. Synthesis of triD$_4$. The products include triD$_4$ and its isomers. All the isomers have similar branch structures and act as triD$_4$.

To the mixture of heptamethylcyclotetrasiloxane (30 mmol, 8.46g) and trivinylmethylsilane (10 mmol, 1.24g) was added xylene solution of Pt catalyst (10 μl) under stirring. The colorless mixture became viscous and light yellow after 30 min. The obtained product was a mixture of triD$_4$ and its isomers and could be used directly without any purification. ^{1}H NMR (400 MHz, $CDCl_3$, δ ppm): 0.500-0.342 (m, 12H, CH_2), 0.100 (s, 63H, CH_3), -0.076 (s, 3H, CH_3). The isomer showed peaks at 1.0 ppm (d, J = 12 Hz, CHCH_3 in an isomer) and the integration of the peaks suggested an isomer/triD$_4$ ratio of 0.23/1.

2.2 *N,N`-bis(3-heptamethylcyclotetrasiloxane-yl-propyl)-perylenediimide (PDI-based dye crosslinker)*

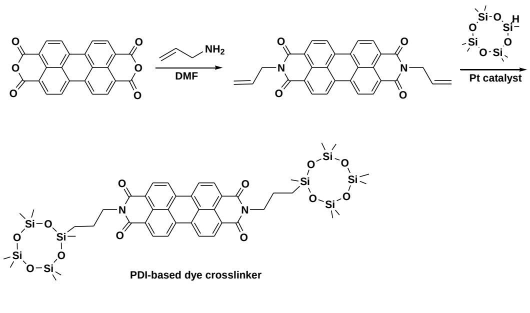

Supplementary Figure 4. Synthesis of PDI-based dye crosslinker. The structure of the labeled crosslinker allows for covalently bonding PDI dye into the polymer matrix.

N,N`-diallyl perylenediimide: To the solution of perylene-3,4,9,10-tetracarboxylic dianhydride (0.1 mmol, 39 mg) in 20 ml dried DMF at 60 °C was added allylamine (10 mmol, 75 μl) dropwise under stirring. After stirring for 1 hour at 60 °C, the mixture was heated to 150 °C for 12 hours. An amaranthine crystal formed as the solution cooled down to rt. The crystal was collected and washed with isopropanol to give 38.5 mg product with a yield of 82 %. ^{1}H NMR (400 MHz, $CDCl_3$, δ ppm): 8.73 (d, 4H, J = 6 Hz, PDI), 8.67 (d, 4H, J = 7 Hz, PDI), 6.08-5.98 (m, 2H, vinyl), 5.37 (d, 2H, J = 13 Hz, vinyl), 5.25 (d, 2H, J = 8 Hz, vinyl), 4.85 (d, 4H, J = 5 Hz, CH_2).

PDI-based dye crosslinker: The mixture of N,N`-diallyl perylenediimide (0.05 mmol, 23.5 mg), heptamethylcyclotetrasiloxane (0.4 mmol, 118 μl), and xylene solution of Pt catalyst (10 μl) in THF (10 ml) was stirred for 10 hours at rt. Both the temperature (21-25 °C) and the humidity (50%) were constant in the lab. After the solvent was removed under vacuum, the residue was purified by flash chromatography on a silica column with the mixture of dichloromethane and hexane (1/1, v/v) as the eluent and a red solid (47 mg) was obtained as the product with a yield of 91 %. The product can be easily dissolved in chloroform, THF, acetone, and hexane. It also dissolves in D_4 to show a spectrum in which the A_{0-0}/A_{0-1} band intensity ratio of PDI-conjugated D_4 is 1.4:1, a characteristic of non-aggregated PDI derivatives (UV spectrum shown in Supplementary Figure 5). ^{1}H NMR (500 MHz, CDCl3, δ ppm): 8.51 (d, 4H, J = 8 Hz, PDI), 8.34 (d, 4H, J = 7 Hz, PDI), 4.19 (t, 4H, J = 8 Hz, CH_2), 1.86-1.80 (m, 4H, CH_2), 0.71 (t, 4H, J = 8 Hz, CH_2), 0.150-0.091 (m, 42H, CH_3). ^{13}C NMR (160 MHz, CDCl3, δ ppm): 163.04, 134.09, 131.01, 129.03, 125.97, 123.19, 122.74, 29.70, 21.68, 14.59, 0.912, 0.83, 0.81, 0.78, 0.73, 0.70, 0.68, 0.58 (44C). MALDI-MS *m/z*: 1057 [M^+Na] (Calculated for $C_{44}H_{62}N_2O_{12}Si_8$: 1034.25).

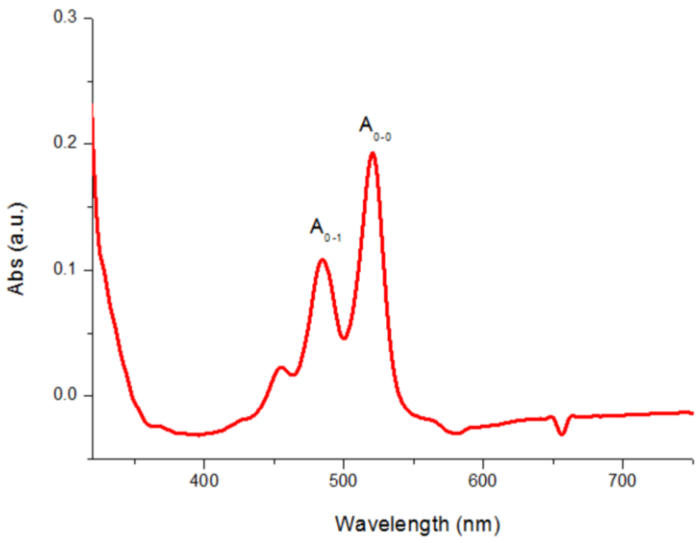

Supplementary Figure 5. UV-vis spectrum of PDI-based dye crosslinker in D_4

Supplementary Note 3: effect of fillers

3.1 *The stabilization of the triflic acid*

Triflic acid is volatile and it could re-form in the matrices and escape from the samples, leading to the deactivation of the monomer-polymer equilibration processes. It is well-known

that the hydrophilic particles whose surfaces have protonatable groups, such as -COOH, -OH, -OR, can firmly absorb organic acids like triflic acid on their surfaces through electrostatic interactions (Supplementary Figure 6)[5]. Carbon black (CB) and silica particles (SiO_2) were selected to show that such interactions could stabilize the acidic species in the living siloxane elastomer matrix. We had confirmed the absorption of acid molecules on CB by the change in zeta potential in a previous study[6]. Briefly, carbon black particles were first dispersed in toluene, and then triflic acid was added to the suspending mixture to allow the absorption of the acid onto the particle surface. After three-time rinsing with toluene and drying, the obtained CB particles were dispersed in water for the zeta potential measurement (0.125 mg/ml). The control sample is the CB particles dispersed in the toluene. The zeta potential for the acid-treated CB is 3.77 ± 0.70 mV, while that of control CB is −27.4 ± 0.69 mV. The same measurement was also conducted to confirm the absorption of acidic molecules on SiO_2. It was found that the zeta potentials for the acid-treated SiO_2 and the control SiO_2 are 6.79 ± 1.01 mV and -6.16±0.69 mV, respectively. The change toward positive value indicated the absorption of positively charged species (acidic species here).

The CB/siloxane and SiO_2/siloxane polymer composites were prepared by curing the mixture of filler, D_4, and triD_4 in the presence of triflic acid. Typically, 1 wt% filler and 1 wt% triflic acid were used in this study. To further confirm the stabilization of fillers to acid species in composites, we compared the stability of acid molecules in CB/siloxane composites and siloxane elastomers. Both kinds of samples were allowed to be exposed to air for 15 days before measurement. As shown in Supplementary Figure 7, the elements sulfur and fluorine from adsorbed triflic acid were observed in the EDX spectrum of CB/siloxane composite but not in the spectrum of filler-free siloxane elastomer. The absence of the elements sulfur and fluorine suggested that the acidic molecules had completely leached away in the test condition while the presence of these elements revealed that fillers like CB could effectively stabilize the acidic species via electrostatic interactions.

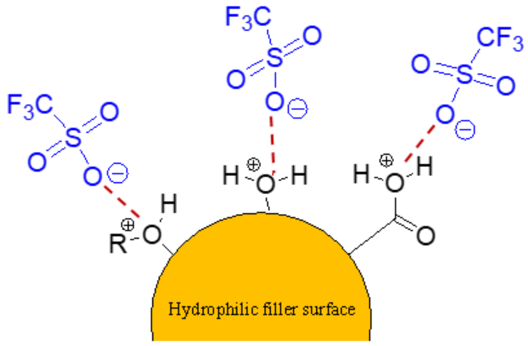

Supplementary Figure 6. Electrostatic interactions between triflic acid and hydrophilic fillers.

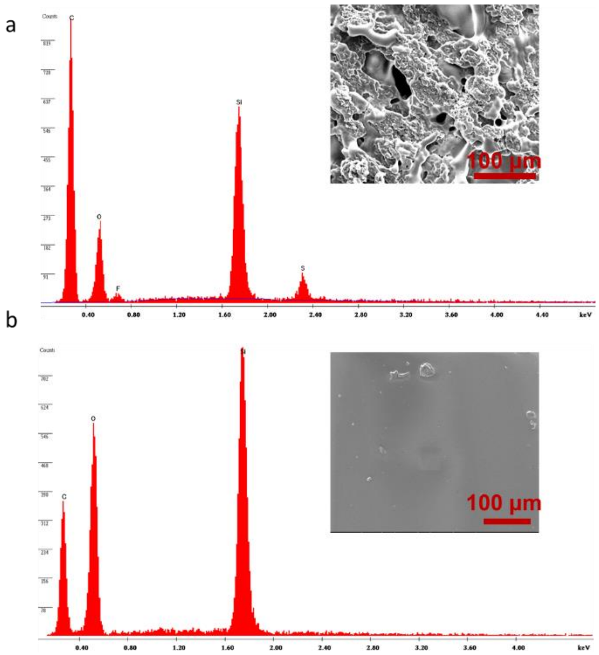

Supplementary Figure 7. EDX spectra of treated CB/siloxane composite (a) and siloxane elastomer (b).

To further prove the stabilization of trifluoromethanesulfonic acid on fillers, we used acid-treated CB to prepare living siloxane elastomer. The acid-treated CB was prepared as follows: CB (0.02 g) was first dispersed in toluene (10 ml) by sonication for 10 minutes. To this mixture, triflic acid (60 μl) was added slowly over 20 minutes under sonication. After the solvent was removed by reduced pressure distillation, a black solid was obtained, which was washed with toluene and then dried at RT for 24h. Such acid-treated CB could be used as an acid catalyst (1 wt%) to trigger the ring-opening copolymerization of D_4 and triD_4 (1 wt%) to get a composite directly (Supplementary Figure 8), indicating that acids were indeed attached on the surface of CB and also maintained their activity to trigger the monomer-polymer equilibration.

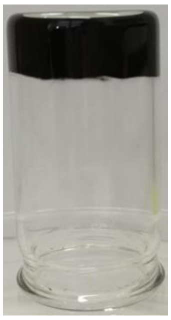

Supplementary Figure 8. CB/siloxane elastomer made from acid-treat CB.

Supplementary Note 4: growth

4.1 *Swelling*

The supplied solution (mixture of D_4 and triD_4) was purged with dry N_2 for 5 min. The as-

prepared samples were immersed in this mixture for swelling. After different immersing times, the samples were taken out and the residual liquid on the surface was removed before measuring their weight. The samples were put back into the mixture solution for further immersion. Normally, eight samples (1×1×0.4 cm) were put together as a group for collecting data (Supplementary Table 1). For the samples used for annealing under an unsaturated swelling state, the swelled samples were stored at rt before annealing in sealed bottles for 12 hours to allow for homogeneous distribution of absorbed liquids throughout the samples. Petri dishes (Supplementary Figure 9) were used for samples' storage, which was sealed by parafilm. All the samples were sealed in this way without further statement.

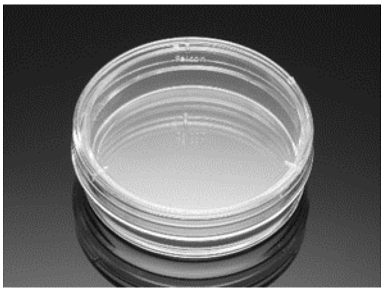

Supplementary Figure 9. Petri dish for sealing samples

To deduce the components of the mixture solution absorbed into the gels, we compared the feed and the residual solutions by using ^{1}H NMR. Two mixture solutions, i.e. the solutions containing 2 wt% and 10 wt% triD_4, respectively, were used and the weight details are shown in Supplementary Table 1. After the complete swelling of the samples, nearly half of the mixture solutions were absorbed and the residual solutions were analyzed by ^{1}H NMR. As shown in Supplementary Figure 10, the peaks highlighted at 1.0, 0.5, and -0.03 ppm are assigned to the protons of triD_4. The spectra of residual solutions completely overlap with those of feed solutions, indicating the same components. Therefore, D_4 and triD_4 were absorbed homogeneously during swelling.

Supplementary Table 1. The weight of samples and mixture solutions (mg)

triD_4 fraction in the feed solution	Starting sample	Feed solution	Swollen sample	Residual solution	triD_4 fraction in residual solution
2.0 wt%	323	2880	1998	1205	2.0 wt%
10.0 wt%	357	2880	1790	1447	10.0 wt%

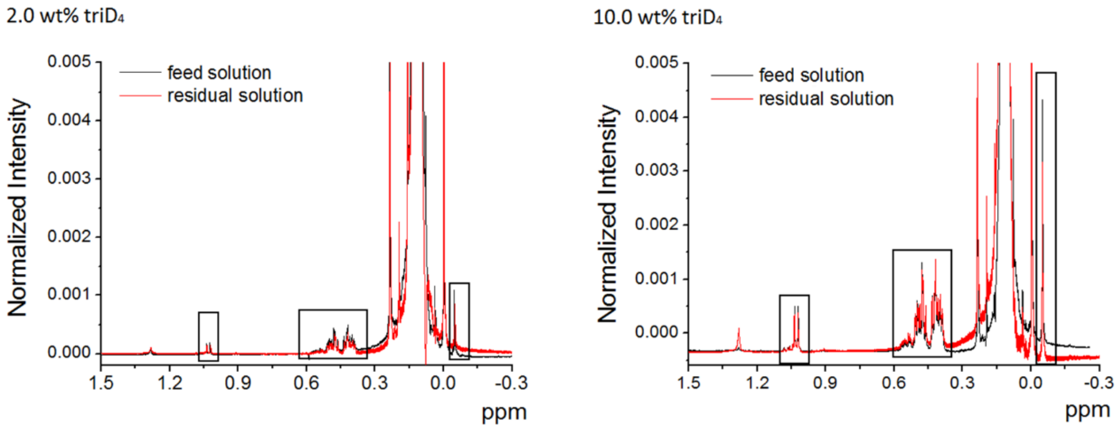

Supplementary Figure 10. ^{1}H NMR spectra of the feed and residual solutions consisting of D_4 and triD_4 in $CDCl_3$.

We assumed that the active living species would not transfer from the sample into the supplied solution during immersion. To confirm this, we annealed the residual solutions at 90 °C for 10 hours. After such annealing, the residual solutions maintained their liquid state without any visible increase in their viscosity. This assumption was also supported by the ^{1}H NMR measurement in which both the annealed and starting solutions show nearly the same spectra.

4.2 *Polymerization*

The swelled samples were taken out from the D_4 and triD_4 mixture solution and then stored in sealed glass bottles at rt (25 °C) for different times. For getting the growth index of the sample at different storage times, the samples after storage were immersed in a hexane solution containing 1 wt% triethylamine to stop the acid-catalyzed reactions and to remove the unreacted reagents. Triethylamine would neutralize the triflic acid and form a salt. To check if the salt can be washed out of the samples, we checked the solubility of the salt in hexane. A salt was prepared at first. Briefly, the triflic acid (100 μl) was added into excess triethylamine (1 ml). The mixture was placed in a hood to allow unreacted triethylamine to evaporate for 6h. The obtained product was further annealed at 90 °C for 3 h to remove residual triethylamine and water, yielding a brown liquid (an ionic liquid, Supplementary Figure 11a) as described in literature[7]. The salt is stable, nonvolatile, and cannot dissolve in hexane (Supplementary Figure 11b). It indicated that the salt would be kept in the polymer matrix. After being immersed for two hours (this time proved sufficient for the samples to become fully swollen), the samples were dried in hood at rt. This washing process was repeated three times and the weights of the dried samples were recorded. To obtain representative data, eight samples were put together and analyzed as a group. As a control, one group of the samples was annealed at 50 °C for 4 hours and a polymerization yield of 96% was obtained (further prolonging the annealing time did not increase the polymerization yield). This method was used to obtain the growth index of the acidic samples (Figure 2b).

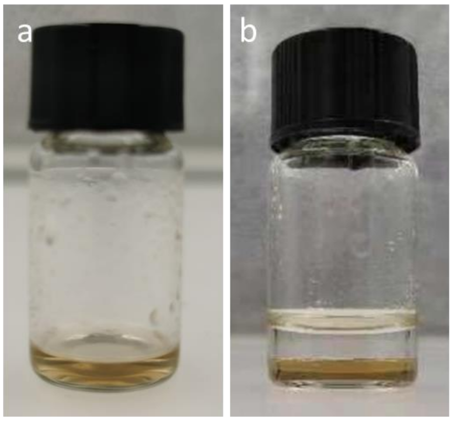

Supplementary Figure 11. (a) The as-prepared salt made from triflic acid and triethylamine and (b) its solubility in the hexane.

We measured the Young's moduli of the swelled samples to monitor the polymerization process and the results are shown in Supplementary Figure 12. The Young's moduli of the samples increased with storage time until 90 hours.

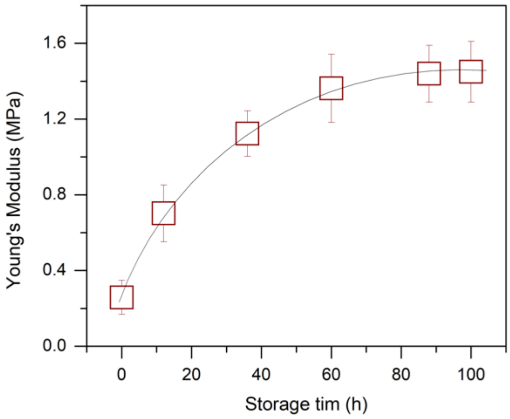

Supplementary Figure 12. Young's modulus of swelled samples stored for different times. The data were obtained from eight independent measurements. Error bars are s.e.m.

4.3 *Uniform growth*

To prove that D_4 and triD_4 mixture solution was integrated homogeneously in the sample, we dyed a starting sample with the PDI-based dye crosslinker and allowed it to grow from a dye-free mixture solution. As shown in Supplementary Figure 13, the dyed living square sample was cut, using a hollow hole puncher, into two parts: a circular piece and a square piece with a circular hole in the center. Both samples were immersed in a mixture solution containing 6 wt% triD_4 for 10 hours and then stored in sealed glass bottles for 4 days, resulting in the grown products. The grown products retain the shape of the original samples. The cross-section under UV irradiation shows a homogeneous emission, indicating a homogeneous expansion of the

original samples.

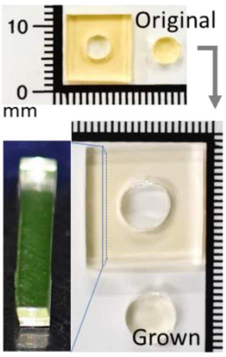

Supplementary Figure 13. The image of a pair of dyed samples and their grown products. The scales in both rulers are the same.

4.4 *The shape and sharp edges change during the growth*

To demonstrate if the samples would retain their sharp edges during the growth, we grew rectangular samples (10 ×10 × 1 mm) with sharp edges (Supplementary Figure 14) to 8 times of their initial weights. Similar sharp edges were observed, as shown in the actual and 3D profiler images.

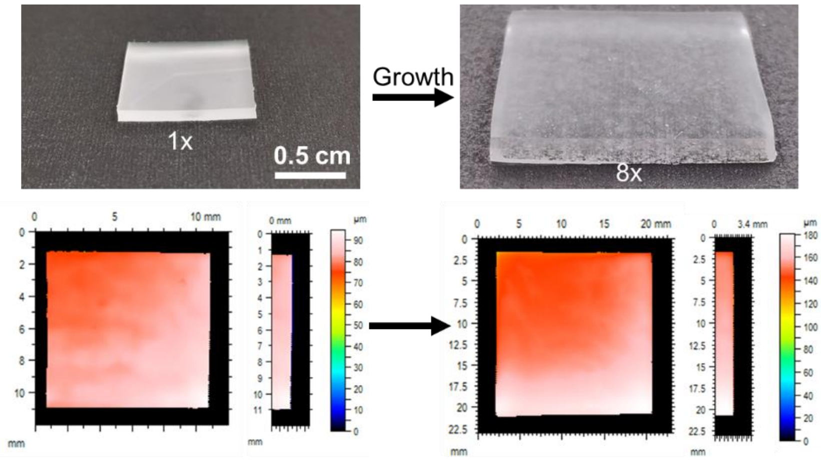

Supplementary Figure 14. The actual and the 3D profiler images showing the shape and the edges of a rectangular sample during the growth process.

4.5 *Growth model*

Due to the constant supply of D_4 and triD_4 mixture solutions and the continuous reaction of chain exchange, the chains kept changing. Additionally, the new chains were formed free of stress while the original chains were stretched. Therefore, the chain end-to-end distance distribution kept changing in the dynamic polymer system. To characterize this dynamic nature of the chains, a transient network model[8] was adopted here with the chains being described with various end-to-end distances in a statistical manner.

Deformation: The original dry network is taken as the reference state. Every material point in

the reference state is assigned with a coordinate $\mathbf{X}$. After deformation, the material point moves to a new position $\mathbf{x}$ in the current configuration. The mapping between the current position and reference position is

$$\mathbf{x} = \mathbf{x}(\mathbf{X}, t). \tag{0.1}$$

The deformation gradient is defined as

$$\mathbf{F} = \frac{\partial \mathbf{x}}{\partial \mathbf{X}}, \tag{0.2}$$

and the volume change of the polymer-monomer composite compared with its reference state is $J = \det \mathbf{F}$.

Incompressibility: The volumes of the individual components including the polymer chains and the free monomers are assumed to be incompressible. At the reference state, only the original network is supposed to exist, therefore,

$$v n_1 C_1 = 1 \tag{0.3}$$

where n_1 is the number of segments per original polymer chain. It is related to the number of Kuhn segments by $n_1 = \alpha n_{1k}$, where α is a constant for a specific type of polymer chain and is dependent on its stiffness. Here, C_1 is the chain density at the reference state and its current concentration is $c_1 = C_1 / J$ and v is the volume of one polymer segment.

After the swelling and polymerization, the material is composed of monomers, the original network, and the new network. The new network and the original network typically have different numbers of segments n. Therefore, the volume of the material is the summation of the polymer and monomer, that is

$$J = 1 + vC_m + vn_2 C_2, \tag{0.4}$$

where C_m is the reference monomer concentration, n_2 is the number of segments per new polymer chain, and C_2 is the density of new chains at the reference state.

Chain distribution: The newly formed dynamic polymer is composed of many polymer chains with different end-to-end distances $\mathbf{r}$. We define the number of chains with end-to-end distance $\mathbf{r}$ per current volume as $\phi(\mathbf{r}, t)$. The reference chain density with end-to-end

distance $\mathbf{r}$ is related to the current density by the relation $\Phi(\mathbf{r},t) = J\phi(\mathbf{r},t)$. The total number of chains per current volume, $c(t)$, satisfies the relation,

$$c(t) = \int_{V^*} \phi(\mathbf{r},t) dV^* = \langle \phi \rangle, \tag{0.5}$$

where V^* is the chain configuration phase space. We also use $\langle \ \rangle$ to represent the integral over all chain configurations. The reference concentration $C(t) = cJ$ is the number of chains per reference volume.

The probability chain density function $P(\mathbf{r},t)$ is defined as

$$P(\mathbf{r},t) = \phi(\mathbf{r},t) / c(t). \tag{0.6}$$

The chain distribution tensor is defined,

$$\boldsymbol{\gamma}(t) = \frac{3}{n_k b^2} \langle P\mathbf{r} \otimes \mathbf{r} \rangle, \tag{0.7}$$

where b is the Kuhn length and n_k is the number of Kuhn segments per chain. Here all chains are assumed to possess the same number of Kuhn segments n_k.

The chain distribution $\phi(\mathbf{r},t)$ can change due to deformation and chemical reactions. The deformation and reaction are assumed to independently influence the evolution of the chain distribution function $\phi(\mathbf{r},t)$,

$$\frac{\partial \phi}{\partial t} = \left.\frac{\partial \phi}{\partial t}\right|_{\text{Deformation}} + \left.\frac{\partial \phi}{\partial t}\right|_{\text{Reaction}}. \tag{0.8}$$

We applied the affine deformation, that is, a chain originally has a chain end-to-end distance $\mathbf{r}_0$ becomes $\mathbf{r} = \mathbf{F}\mathbf{r}_0$ after deformation.

If we take an arbitrary volume V^* in the chain configuration space, the total probability in this space will not change. By applying the Reynolds transport theorem, we would have

$$0 = \frac{d}{dt}\int_{V^*} P dV^* = \int_{V^*} \frac{\partial P}{\partial t} dV^* + \int_{\partial V^*} (\dot{\mathbf{r}} \cdot \mathbf{n}) P dS^*. \tag{0.9}$$

where $\dot{\mathbf{r}} = \mathbf{Lr}$ is the velocity of the chain configuration boundary ∂V^*, $\mathbf{L} = \dot{\mathbf{F}}\mathbf{F}^{-1}$ is the velocity gradient, and $\mathbf{n}$ is the unit norm of the chain configuration boundary ∂V^*.

Applying the divergence law to the Eq. (0.9), we would have the evolution equation of P under deformation as

$$\frac{\partial P}{\partial t} = -\frac{\partial P}{\partial r_i} L_{ij} r_j - P L_{ii}. \tag{0.10}$$

The reference concentration $C(t)$ is constant in the absence of any chemical reaction. From the relation $C = cJ$, the evolution equation for $c(t)$ is

$$\frac{dc}{dt} = -c L_{ii}. \tag{0.11}$$

Combining Eqs. (0.6), (0.10) and (0.11), the evolution equation for ϕ can be obtained as,

$$\frac{\partial \phi}{\partial t} = -\frac{\partial \phi}{\partial r_i} L_{ij} r_j - 2\phi L_{ii}. \tag{0.12}$$

The evolution equation for $\phi(\mathbf{r}, t)$ due to reaction under chain association and dissociation is

$$\frac{\partial \phi}{\partial t} = \xi_a P^0(\mathbf{r}) - \xi_d \phi(\mathbf{r}), \tag{0.13}$$

where ξ_a is the number of chains associated per current volume per unit time, and ξ_d is the dissociation reaction rate constant. It is assumed that the new chains are generated in a stress-free state, so their probability distribution function $P^0(\mathbf{r})$ follows the Gaussian distribution,

$$P^0(\mathbf{r}) = \left(\frac{3}{2\pi n_k b^2} \right)^{3/2} \exp\left(-\frac{|\mathbf{r}|^2}{2 n_k b^2 / 3} \right), \tag{0.14}$$

and it is easy to verify that

$$\mathbf{I} = \frac{3}{n_k b^2} \left\langle P^0 \mathbf{r} \otimes \mathbf{r} \right\rangle. \tag{0.15}$$

Combining the results Eq. (0.12) and (0.13), we have

$$\frac{\partial \phi}{\partial t} = \xi_a P^0(\mathbf{r}) - \xi_d \phi - \frac{\partial \phi}{\partial r_i} L_{ij} r_j - 2\phi L_{ii}. \tag{0.16}$$

Integrating Eq. (0.16) over all chain configurations, we would obtain

$$\frac{dc}{dt} = \xi_a - \xi_d c - c L_{ii}. \tag{0.17}$$

Then we would obtain the evolution equation of P by the relation Eq. (0.6)

$$\frac{\partial P}{\partial t}=\frac{\xi_a}{c}\left(P^0-P\right)-\frac{\partial P}{\partial r_i}L_{ij}r_j-PL_{ii}. \qquad (0.18)$$

Then the time derivative of $\boldsymbol{\gamma}(t)$ becomes

$$\frac{d\boldsymbol{\gamma}}{dt}=\frac{3}{n_k b^2}\int\frac{\partial P}{\partial t}\mathbf{r}\otimes\mathbf{r}dV^*=\frac{\xi_a}{c}(\mathbf{I}-\boldsymbol{\gamma})+\boldsymbol{\gamma}\mathbf{D}+\mathbf{D}\boldsymbol{\gamma}, \qquad (0.19)$$

where $\mathbf{D}=\frac{1}{2}\left(\mathbf{L}+\mathbf{L}^{\mathrm{T}}\right)$.

In the system, there are two reactions, polymerization and chain exchange reaction. As for polymerization, the reaction rate is

$$\xi_{a,p}=k_p c_m c_a/n, \qquad (0.20)$$

where k_p is the polymerization reaction rate constant, c_m is the current concentration of the monomers, c_a is the current concentration of catalysts, and n is the number of segments of the polymerized chains.

The chain exchange reaction can be treated as two reactions at the same rate. One is the chain association and the other is chain dissociation. Their reaction rates are

$$\xi_{a,e}=k_e c, \qquad (0.21)$$

$$\xi_d=k_e. \qquad (0.22)$$

As a result, the overall association rate is

$$\xi_a=\xi_{a,p}+\xi_{a,e}=k_p c_m c_a/n+k_e c. \qquad (0.23)$$

In the experiment, the new network forms in the existence of the original network. The two networks evolve independently and contribute to the elastic stress separately. The original network only participates in the chain exchange reaction, so the evolution equations of the quantities related to the first network ϕ_1, c_1 and γ_1 can be obtained directly from Eq.(0.16), (0.17) and (0.19)

$$\frac{\partial\phi_1}{\partial t}=k_e c_1 P^0(\mathbf{r})-k_e\phi_1-\frac{\partial\phi_1}{\partial r_i}L_{ij}r_j-2\phi_1 L_{ii}, \qquad (0.24)$$

$$\frac{dc_1}{dt}=-c_2 L_{ii}, \qquad (0.25)$$

$$\frac{d\boldsymbol{\gamma}_1}{dt}=k_e(\mathbf{I}-\boldsymbol{\gamma}_1)+\boldsymbol{\gamma}_1\mathbf{D}+\mathbf{D}\boldsymbol{\gamma}_1. \tag{0.26}$$

For the second network, it participates in both reactions, so the evolution equations are

$$\frac{\partial \phi_2}{\partial t}=\left(\frac{k_p c_m c_a}{n_2}+k_e c_2\right)P^0(\mathbf{r})-k_e\phi_2-\frac{\partial \phi_2}{\partial r_i}L_{ij}r_j-2\phi_2 L_{ii},$$

(0.27)

$$\frac{dc_2}{dt}=k_p c_m c_a / n_2 - c_2 L_{ii}, \tag{0.28}$$

$$\frac{d\boldsymbol{\gamma}_2}{dt}=\left(\frac{k_p c_m c_a}{n_2 c_2}+k_e\right)(\mathbf{I}-\boldsymbol{\gamma}_2)+\boldsymbol{\gamma}_2\mathbf{D}+\mathbf{D}\boldsymbol{\gamma}_2. \tag{0.29}$$

Governing equations: The mass conservation equation is first considered. The material is composed of a polymer network and monomers diffusing in the network. The reference concentration of monomer is C_m. The mass conservation equations in the reference configuration for monomers is

$$\frac{dC_m}{dt}+\mathrm{Div}\mathbf{J}_m=-k_p C_m C_a / J, \tag{0.30}$$

where $\mathbf{J}_m$ denotes the nominal flux of monomers.

The catalysts can percolate through the network, so the current concentration of the catalyst is constant. Due to the mass conservation of the catalyst, we would have

$$c_a V = c_{a0} V_0 \tag{0.31}$$

where V_0 and V is the initial overall volume and current overall volume, respectively, and c_{a0} is the initial catalyst concentration

Apart from the mass conversation equation, linear and angular momentum balance equations are also checked. Since the motion of the material is slow, the inertia effect can be ignored, so the linear momentum balance gives

$$\mathrm{Div}\mathbf{P}^{\mathrm{T}}=0, \tag{0.32}$$

where $\mathbf{P}(\mathbf{X},t)$ is the first Piola-Kirchhtress tensor. The Cauchy stress $\boldsymbol{\sigma}$ is related to $\mathbf{P}$ by $\boldsymbol{\sigma}=J^{-1}\mathbf{P}\mathbf{F}^{\mathrm{T}}$. The angular momentum balance gives

$$\mathbf{P}\mathbf{F}^{\mathrm{T}}=\mathbf{F}\mathbf{P}^{\mathrm{T}}. \tag{0.33}$$

Constitutive relations: The elastic force of one chain with the end-to-end vector $\mathbf{r}$ is

$$f(\mathbf{r})=\frac{kT}{b}\mathrm{L}^{-1}\left(\frac{|\mathbf{r}|}{n_k b}\right), \tag{0.34}$$

where k is the Boltzmann constant, T is the absolute temperature, $\mathrm{L}(x)=\coth(x)-1/x$ is the Langevin function, and L^{-1} is its inverse function. The elastic stress is the summation of the force of every chain, which can be obtained from

$$\boldsymbol{\sigma}=\frac{kTc}{b}\left\langle\left(P(\mathbf{r})-P^0(\mathbf{r})\right)\mathrm{L}^{-1}\left(\frac{|\mathbf{r}|}{n_k b}\right)\frac{1}{|\mathbf{r}|}\mathbf{r}\otimes\mathbf{r}\right\rangle. \tag{0.35}$$

To get rid of the integration over all chain configurations, we take $\mathrm{L}^{-1}\left(\frac{|\mathbf{r}|}{n_k b}\right)\frac{1}{|\mathbf{r}|}$ out of the average and assume

$$\boldsymbol{\sigma}=\frac{kTc}{b}\mathrm{L}^{-1}\left(\frac{\bar{r}}{n_k b}\right)\frac{1}{\bar{r}}\langle P(\mathbf{r})\mathbf{r}\otimes\mathbf{r}\rangle-\frac{kTc}{b}\mathrm{L}^{-1}\left(\frac{\bar{r}_0}{n_k b}\right)\frac{1}{\bar{r}_0}\left\langle P^0(\mathbf{r})\mathbf{r}\otimes\mathbf{r}\right\rangle, \tag{0.36}$$

where $\bar{r}$ and $\bar{r}_0$ is the average chain length under probability function $P(\mathbf{r})$ and $P^0(\mathbf{r})$, respectively. The average chain lengths are

$$\bar{r}=\sqrt{\left\langle P|\mathbf{r}|^2\right\rangle}=\sqrt{\frac{n_k b^2}{3}\mathrm{tr}\boldsymbol{\gamma}}, \tag{0.37}$$

$$\bar{r}_0=\sqrt{\left\langle P^0|\mathbf{r}|^2\right\rangle}=\sqrt{n_k}b. \tag{0.38}$$

and the stress is

$$\boldsymbol{\sigma}=\frac{kTc\sqrt{n_k}}{3}\left(\mathrm{L}^{-1}\left(\sqrt{\frac{\mathrm{tr}\boldsymbol{\gamma}}{3n_k}}\right)\sqrt{\frac{3}{\mathrm{tr}\boldsymbol{\gamma}}}\boldsymbol{\gamma}-\mathrm{L}^{-1}\left(\sqrt{\frac{1}{n_k}}\right)\mathbf{I}\right) \tag{0.39}$$

Consider the special case of two networks, the stress should be the summation of the two networks. It is necessary to add the osmotic pressure induced by free monomers and ions. In addition, when the Cohen's approximation is applied

$$\mathrm{L}^{-1}(x)=x\frac{3-x^2}{1-x^2}. \tag{0.40}$$

then have

$$\boldsymbol{\sigma}=\frac{kTc_1}{3}\left(\frac{9n_{1k}-\mathrm{tr}\boldsymbol{\gamma}_1}{3n_{1k}-\mathrm{tr}\boldsymbol{\gamma}_1}\boldsymbol{\gamma}_1-\frac{3n_{1k}-1}{n_{1k}-1}\mathbf{I}\right)+\frac{kTc_2}{3}\left(\frac{9n_{2k}-\mathrm{tr}\boldsymbol{\gamma}_2}{3n_{2k}-\mathrm{tr}\boldsymbol{\gamma}_2}\boldsymbol{\gamma}_2-\frac{3n_{2k}-1}{n_{2k}-1}\mathbf{I}\right)+\Pi\mathbf{I}, \quad (0.41)$$

where Π is the osmotic pressure induced by free monomers.

The chemical potential of the monomers can be obtained from the previous reference

$$\mu_m=kT\left(\ln\frac{C_m}{C_m+nC}+\frac{nC}{C_m+nC}+\chi\frac{n^2C^2}{\left(C_m+nC\right)^2}\right)-v\Pi, \quad (0.42)$$

The flux of the monomer follows the linear relation in the current configuration

$$\mathbf{j}_m=-\frac{D_m c_m}{kT}\frac{\partial\mu_m}{\partial\mathbf{x}}, \quad (0.43)$$

where D_m is the diffusivity of the monomers. The diffusivity is assumed to be proportional to the monomer volume fraction $\frac{vC_m}{J}$, so $D_m=D_{m0}\frac{vC_m}{J}$ with D_{m0} being a constant.

The nominal flux and the current flux are related through the relation

$$\mathbf{J}=J\mathbf{F}^{-1}\cdot\mathbf{j}. \quad (0.44)$$

So the fluxes in the reference configurations are

$$\mathbf{J}_m=-\frac{vC_m}{J}\frac{D_{m0}C_m}{kT}\mathbf{F}^{-1}\mathbf{F}^{-\mathrm{T}}\frac{\partial\mu_m}{\partial\mathbf{X}} \quad (0.45)$$

The parameters using in the experiment fitting are,

	Value	Unit	Description
v	10^{-28}	m^3	Volume of one molecule
vC_1	1/14	Dimensionless	Number of polymer chains per monomer
n_1	14	Dimensionless	Number of segments per original chain
α	2.5	Dimensionless	Number of segments per Kuhn segment
n_2	30	Dimensionless	Number of segments per new chain
k_p	6×10^{-5}	$1/\mathrm{s}$	Polymerization reaction coefficient
k_e	6×10^{-6}	$1/\mathrm{s}$	Chain exchange reaction coefficient

χ	0	Dimensionless	Flory–Huggins interaction parameter
D_{m0}	4×10^{-10}	m^2/s	Diffusivity of monomers

We first used the deactivated swelling data to get the equilibrium swelling ratio and the diffusion rate (black squares and black line in Figure 2a). When the material is deactivated, all chemical reaction rates are zero. The polymer swells from its initial state (Swelling ratio = 25/24) to the equilibrium state. From the equilibrium swelling ratio, vC_1, n_1, α can be fitted. Since the polymer and monomer are the same species, it is assumed that the interaction between monomers and monomers and polymer segments are the same. As a result, χ should be equal to 0. The swelling rate depends on diffusion coefficients, D_{a0}.

Then, we fitted reaction constants k_p and k_e from second experiments, in which the polymer still swells from the same initial state as the first experiment, but since the monomers are polymerized, more monomers tend to diffuse into the new network (green circles and green line in Figure 2b). In addition, the stress in the network would be relaxed due to the chain exchange reaction. The swelling ratio difference between these two experiments provides enough information to extract the reaction constants.

In the end, we used the parameters to compute the theoretical results of polymerization experiments. In the experiments, the material is first put into the monomer for 10 hours and then sealed in the bottom for 90 hours. As a result, we applied the constant monomer chemical potential boundary at the first 10 hours and the no-flux boundary for the remaining 90 hours. The theoretical results are compared with the experiment data (orange circles and the solid line in Figure 2a). The theoretical results and the experiments fit well, which suggests the theory is valid.

4.6 *Mechanical properties of acid-activated growth samples*

The Young's modulus was obtained by compression measurements (Instron Model 5566). The samples (1(w)×1(l) ×0.4(h) cm) were compressed 25% to get their moduli (Supplementary Table 2). Eight independent samples were measured as a group for data collection. Typical acidic samples (made from a solution containing 2 wt% triD_4) were prepared and immersed in the mixture solution with different triD_4 concentrations for either 6 or 10 hours. After their removal from the mixture solution, the samples were sealed and stored at rt for one week before measuring their moduli (the grown products were cut into the specific size for measuring). As controls, samples made from 2 wt% and 6 wt% triD_4, were measured respectively. They showed Young's moduli of 1.67±0.12 and 2.74±0.17 MPa, respectively.

Supplementary Table 2. Growth index and Young's modulus of grown samples obtained under

different conditions (Growth index: $W_{grown}/W_{original}$, Error bars are s.e.m.).

$triD_4$ in mixture solution (wt%)	Growth index for six-hour immersion	Young's Modulus (MPa)	Growth index for ten-hour immersion	Young's Modulus (MPa)
2	5.02±0.88	1.51±0.06	6.58±1.04	1.44±0.07
4	5.17±0.84	1.59±0.05	6.64±0.64	1.50±0.06
6	4.8±0.7	1.61±0.05	6.25±0.64	1.59±0.07
8	5.04±0.48	1.68±0.07	6.33±0.88	1.65±0.06
10	4.4±0.88	1.72±0.06	5.91±1.12	1.70±0.07

Supplementary Note 5: degrowth

5.1 *Degrowing mechanism and the effect of stabilizer.*

The evaporation-induced depolymerization mechanism (Supplementary Figure 15a) was investigated. Supplementary Figure 15b shows the setup used to collect the volatile components that escaped from the materials. Note that this setup was only used for collecting the liquid for component study since the reduced pressure favored the evaporation of bigger cyclic molecules. The degrowth of the samples could be conducted at rt in air. The set-up was connected to a vacuum pump to generate reduced pressure (~10 mbar) to induce the evaporation of the small cyclic compounds generated in depolymerization. These compounds were condensed in the trap which was cooled with liquid nitrogen. Under this condition, the sample loaded in the container lost about 25 % of its weight in 3h (Supplementary Figure 15c). By contrast, a deactivated sample would only lose 6-10 % of its weight after washed with hexane. The extra decrease in weight was attributed to depolymerization.

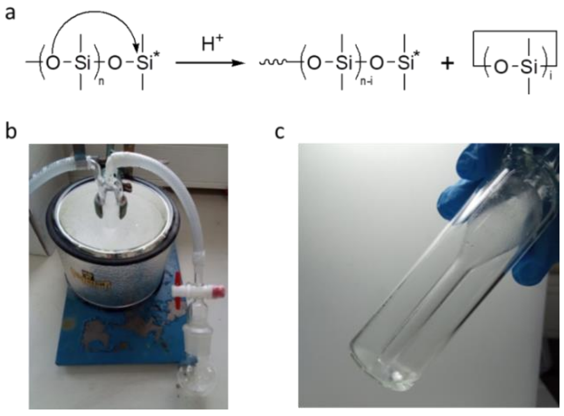

Supplementary Figure 15. Evaporation-induced degrowth. a) Backbiting reaction. b) A set-up used for the collection of the evaporated liquid. c) The collected liquid in the trap.

The collected liquid was analyzed by ^{1}H NMR and GC-MS (Supplementary Figure 16). With pure hexanmethylcyclotrisiloxane (D_3), D_4, and decamethylcyclopentasiloxanes (D_5) as standards, we confirmed by ^{1}H NMR results that the liquid collected from living CB/PDMS elastomers consisted of D_4 (78 mol%), D_5 (20 mol%), and a trace amount of D_3 while that obtained from living PDMS elastomers contained similar components, i.e. D_4 (81 mol%), D_5 (16 mol%) and a trace amount of D_3. Similar results were observed from GC-MS analysis: the liquid generated in the composite sample was comprised of D_4 (80.8 mol%), D_5 (17.6 mol%), and D_6 (1.6 mol%) while the liquid collected from the filler-free sample consisted of D_4 (85.5 %) and D_5 (14.5%). These results supported the backbiting depolymerization mechanism and also suggested that the filler did not quench the activity of the acidic species on the depolymerization.

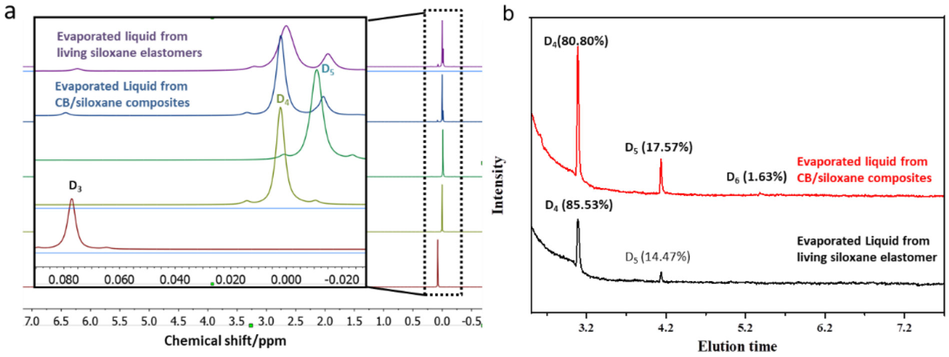

Supplementary Figure 16. Characterization of collected liquid. a) ^{1}H NMR spectra of comparison of the collected liquids obtained from different samples D_3, D_4, D_5, and the evaporated oligomer mixture from the living siloxane elastomer and CB/siloxane composites. b) GC-MS curves of the evaporated oligomer mixture from the living siloxane elastomers and CB/siloxane composites.

The fillers could stabilize the acidic species and therefore, their addition favored the degrowth. As shown in Supplementary Figure 17, the degrowth rate increased with the fraction of fillers (CB or SiO_2).

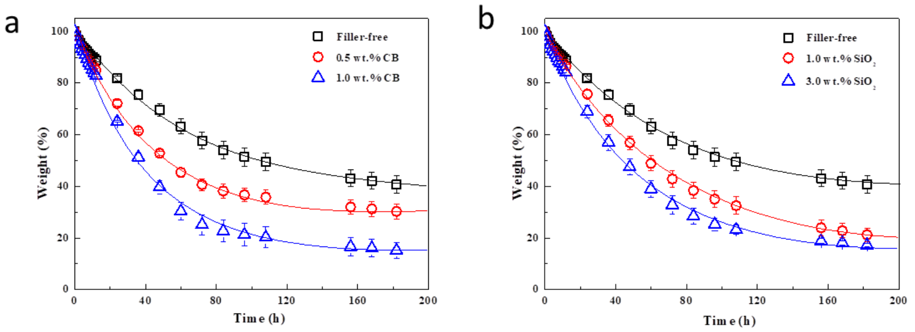

Supplementary Figure 17. The degrowing process of the siloxane composites with different filler contents. a) carbon black, b) SiO_2. The data were obtained from eight independent measurements. Error bars are s.e.m.

5.2 *Degrowth model*

At the reference state, the reference chain density C_1 and the number of segments per chain n_1 satisfies relation (0.3). When the network degrows, the chain density does not change but every chain shortens to n_t, so the overall volume of the dry networks is vn_tC_1 and the incompressibility condition becomes

$$J = vn_tC_1 + vC_m, \tag{0.31}$$

In the dry state, the chain is in its natural configuration and its length is $\sqrt{n_1/\alpha}b$ in the original network and $\sqrt{n_t/\alpha}b$ in the degrown network. When these two networks reach the same deformation gradient, $\mathbf{F}$, the degrown network is stretched $\sqrt{n_1/n_t}$ longer than the original network, so the elastic deformation gradient for the degrown network $\mathbf{F}^e$ is related with the apparent deformation gradient $\mathbf{F}$ by the relation

$$\mathbf{F}^e = \sqrt{\frac{n_1}{n_t}}\mathbf{F}. \tag{0.32}$$

The free energy of the system is composed of elastic energy of the network and mixing energy between polymers and monomers,

$$W = \frac{1}{2}C_1kT\left(\mathbf{F}^e : \mathbf{F}^e - 3 - 2\ln J^e\right) + \frac{kT}{v}\left[vC_m \ln\left(\frac{C_m}{n_tC_1 + C_m}\right) + \chi\frac{vn_tC_1C_m}{n_tC_1 + C_m}\right] + \Pi\left(J - vn_tC_1 - vC_m\right)$$

(0.33)

The last term represents the incompressibility constraint.
With the free energy function Eq. (0.33), we can derive the constitutive relations. The first Piola-Kirchhoff stress is

$$\mathbf{P} = \frac{\partial W}{\partial \mathbf{F}} = C_1kT\left(\frac{n_1}{n_t}\mathbf{F} - \mathbf{F}^{-T}\right) + \Pi J\mathbf{F}^{-T}.$$

(0.34)

The chemical potential of monomer is

$$\mu_m = \frac{\partial W}{\partial C_m} = kT\left[\ln\left(1 - \frac{vn_tC_1}{J}\right) + \frac{vn_tC_1}{J} + \chi\frac{\left(vn_tC_1\right)^2}{J^2}\right] - \Pi v.$$

(0.35)

The degrowth reaction rate is proportional to the number of monomers in the network per

current volume and also depends on the concentration of the catalyst. In the meantime, the reverse reaction in which the monomers are also added back to degrown network also proceeds. Therefore, the number of cyclic molecules (monomer) generated per current volume is

$$\xi = k_d \frac{n_t C_1}{J} c_a^{\beta} - k_{db} \frac{C_m}{J} c_a^{\beta} \tag{0.36}$$

where k_d is the reaction rate constant, k_{db} is the backward reaction rate constant, and β is the reaction order of catalyst.

As a result, the mass conservation for the network is

$$\frac{d}{dt}(n_t C_1) = -J\xi , \tag{0.37}$$

and the mass conservation for monomers is

$$\dot{C}_m + \mathrm{Div}\,\mathbf{J}_m = J\xi . \tag{0.38}$$

When the chain becomes short, the mesh size of the network is small. As a result, the monomer diffusivity is reduced. It is assumed that D_{m0} depends on chain length through the scaling behavior

$$D_{m0} = D(n_t / n_1)^{\gamma} . \tag{0.39}$$

In the experiments, there are two types of degrowth scheme, degrowth filler-free or with filler. Initially, the catalyst concentration c_{a0} is a constant in the material. In the first scheme, the catalyst can percolate fast in the sample, so it is assumed the current concentration is still constant throughout the material. Due to the mass conservation of the catalyst, we would have

$$c_a V = c_{a0} V_0 \tag{0.40}$$

where V_0 and V are the initial overall volume and current overall volume, respectively. The catalyst can evaporate from the material. Consequently, the material's volume will reach a constant when all the catalysts are evaporated. The evaporation rate per area is assumed to be hc_a where h is the evaporation rate coefficient and c_a is the catalyst concentration on the surface.

As for the second scheme, the catalyst is absorbed by the fillers, so the reference concentration at each point will not change,

$$c_a(\mathbf{x}) J(\mathbf{x}) = c_{a0}(\mathbf{X}) J_0(\mathbf{X}) \tag{0.41}$$

The parameters using in the experiment fitting are,

	Value	Unit	Description

β	2	Dimensionless	Reaction order of catalyst
γ	2.5	Dimensionless	Diffusivity dependence on chain length
k_d	2.5×10^{-6}	$1/s$	Degrowth reaction coefficient
k_{db}	$5k$	$1/s$	Reverse reaction coefficient
h	9.5×10^{-10}	m/s	Evaporation rate coefficient

All other unlisted parameters are the same as the previous table for growth modeling.

In the experiment, the material is exposed to the air so that monomers can evaporate from it. Therefore, $\mu_m = -3kT$ is applied on the boundary to drive the monomer outside. Further reducing the chemical potential on the boundary will not influence the results. The computational results fit well with the experiments.

5.3 *On-off control of the degrowth.*

Typical living siloxane elastomers (2 g) were allowed to degrow at rt in air for 12 h (lost 11 wt%). The degrowth was quenched by the triethylamine deactivation treatment described in Methods section and then deactivated samples were obtained. These samples were stored at rt in air for 1 day for evaluating their stabilities. The samples were reactivated by dropping 20 μl triflic acid directly on the surface of the deactivated materials. It was observed that the liquid (triflic acid) was absorbed by the material in 30 min. Four on-off degrowth cycles were conducted and they led to similar weight loss (11, 8, 8, and 7 wt% for the first, second, third, and fourth cycles, respectively (Figure 3b).

Since triethylamine was used to deactivate the samples, it could be argued that there was triethylamine residue in the materials. These residual molecules would neutralize the acid catalyst that was added into the sample for turning on the growth/degrowth. Therefore, it was necessary to check the triethylamine residue in the deactivated sample. Briefly, a living siloxane was first deactivated by immersing in TEA. The deactivated sample was then taken out of TEA and stored in a hood. FT-IR spectroscopy was used to monitor the sample after the different evaporation times, with living siloxane and TEA as references. As shown in Supplementary Figure 18a, the peak at around 1204 cm^{-1} was assigned to the C-N stretching of TEA. The peak is available in the fresh deactivated sample. It is not detectable after 2h-evaporation. These results implied that the free TEA had completely escaped out of the samples.

We also tested the long-term stability of the deactivated siloxanes by annealing the deactivated samples in an oven at 100 °C for one week. The weight loss is negligible (<1 wt%), indicating that the deactivated samples was stable.

The residual salts within the deactivated samples could be removed by washing with tetrahydrofuran (THF). Briefly, the samples were immersed in THF for 30 minutes, followed by drying in a fume hood for 12 hours for solvent evaporation. This process was repeated three times to ensure thorough removal of the salts. ATR-FTIR spectroscopy was used to monitor the washing process. As shown in Supplementary Figure 18b, the as-prepared deactivated samples

showed a characteristic peak at around 1226 cm^{-1}, which was attributed to the C-F stretching of the salts. After washing, this peak disappears, indicating that the salts could be effectively removed from the samples.

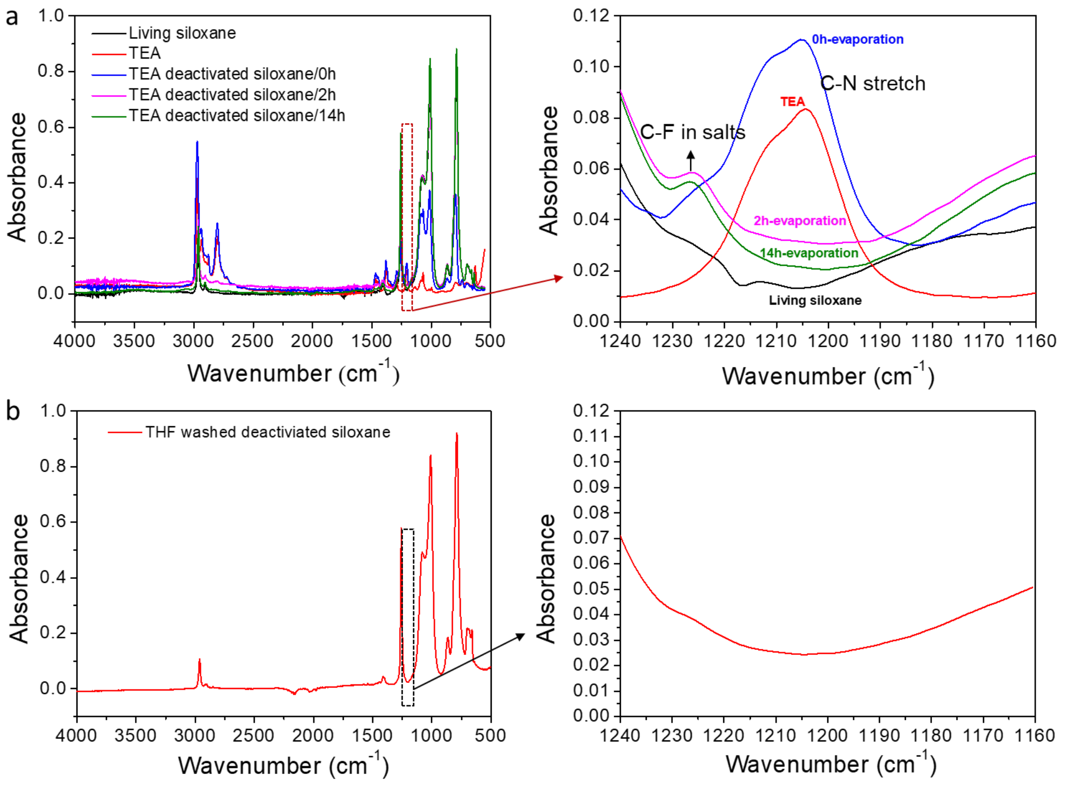

Supplementary Figure 18. (a) ATR-FTIR spectra of the living siloxane, triethylamine, deactivated siloxane at different evaporation times. (b) ATR-FTIR spectrum of the THF washed deactivated siloxane.

5.4 *Self-stiffening during the degrowth*

It was assumed that the crosslinker would not evaporate during the degrowth and therefore, the crosslinking degree would increase with degrowth. To confirm this, a living siloxane elastomer was first degrown to 30 wt% of its initial weight. It was found that the modulus of the degrown sample increased (Supplementary Figure 19). Moreover, when the degrown sample was re-grown from a triD_4-free solution to the initial weight at rt for 7 days. The E-modulus of the re-grown composite was comparable to the as-prepared elastomers (both are 260 ± 19 KPa), indicating that the crosslinker did not degrade and evaporate during degrowing.

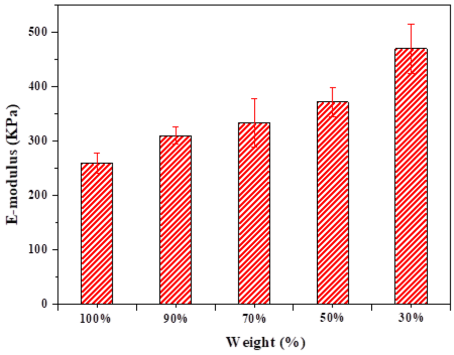

Supplementary Figure 19. Young's moduli of the living siloxane elastomers during the degrowth. The data were obtained by tensile measurements. The data were obtained from eight independent measurements. Error bars are s.e.m.

5.5 *The shape and sharp edges change during the degrowth*

A cubic sample (10 × 10 × 1 mm) was prepared and placed in a hood at rt for degrowth. During the degrowth, we took the sample's photos and measured its 3D profiles to monitor its shape change. As shown in Supplementary Figure 20, the degrown sample maintained its shape overall. The sharp edges maintained their morphologies when 40 wt% of the sample was lost but became rough as the sample degrowed to 60% of the initial weight.

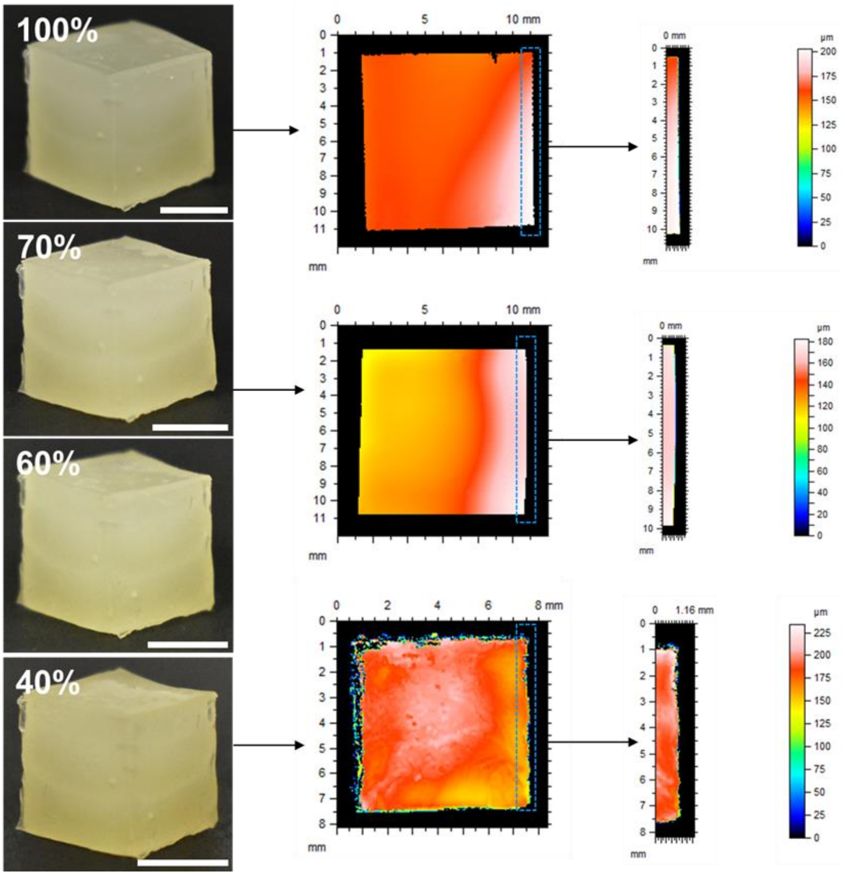

Supplementary Figure 20. The actual and the 3D profiler images showing the shape and edges of a cubic sample in a typical degrowth process. Scale bar is 0.5 cm.

5.6 *Removal of small cyclic molecules from siloxane elastomers*

The removal of small cyclic molecules generated in depolymerization included two steps: diffusion of the molecules from the matrices to the surfaces of materials and the evaporation of the molecules on the surfaces. It is generally accepted that the evaporation of small molecules in crosslinked polymers is a diffusion-controlled process[9]. In other words, the process of small molecule evaporation from the surface to the environment is significantly faster than their diffusion process. A similar process was expected in the siloxane elastomers studied here. Note that the diffusion of molecules from a matrix to a surface was exactly the opposite process of swelling in which molecules diffused from the surface into the matrix[10,11]. Therefore, the diffusion rate could be evaluated by a swelling process. To avoid the contribution of polymerization/depolymerization to the diffusion, deactivated siloxane elastomers were used for the tests. Here a mixture solution of D_4 and D_5 was used to swell the samples because the depolymerization preferred to generate D_4 and D_5. After full swelling, the swollen samples were stored in air at rt (the same condition for degrowth). As shown in Supplementary Figure 21, the samples reach a saturated swelling state in 22 h, implying that the molecules required 22 h to diffuse through the samples. The time for these small molecules to escape from the matrices was also 22 h. The similar values in both processes supported the hypothesis that the removal of small cyclic molecules was a diffusion-controlled process. The process was significantly slower than degrowth.

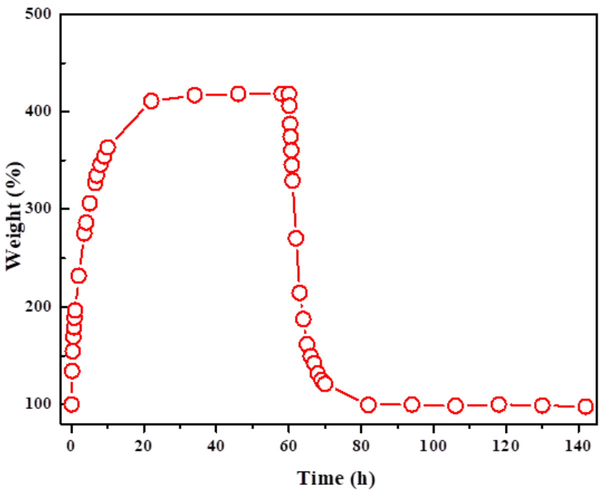

Supplementary Figure 21. Weight change of a deactivated siloxane elastomer in a swelling-evaporation process. A sample with a thickness of 2 mm was used and the mixture used for swelling consisted of 85 wt% D_4 and 15 wt% D_5.

5.7 *Homogenous degrowth for the living siloxane elastomers*

Fluorescence measurements in the dyed living siloxane elastomers: A dyed living siloxane elastomer was prepared to demonstrate the uniform structure formed via the homogeneous degrowth in the absence of fillers. Typically, a dyed living siloxane sample was cut into three pieces. One was used as the reference (as-prepared sample); one was allowed to degrow to 50 % of its initial weight (degrown sample); the last one was first degrown to 50% of the initial weight and then regrown back to its initial weight (regrown sample). The solution used for

regrowing the degrown samples was normal D_4. The degrowth was performed in air by placing the sample on a petri dish where the evaporation occurred only on the top surface, rather than the bottom surface. To regrow the degrown sample, the mixture solution was dropped to the degrown sample (the liquid was absorbed soon, rather than concentrated on the surface), followed by storage in a sealed plastic bottle for growth. This method allowed us to control the growth index. Fluorescence confocal microscopy was used to study the structure. The cross-section images of the samples at different states were collected to get the fluorescence intensities (Figure 3d and Figure 3fi). It was observed that the fluorescence intensities at different z-positions were nearly the same for the as-prepared, degrown, and regrown samples, indicating homogeneous matrices. Moreover, the fluorescence intensity increased for the degrown samples because the dye concentration increased with degrowth as a consequence of the nonvolatile nature of the dye crosslinker. When the sample grew back to its original weight from a dye-free solution, the fluorescence density turned back to its original level.

5.8 *Heterogeneous degrowth of the living filler/siloxane composites*

Fluorescence measurements in the dyed filler/siloxane composites: A dyed SiO_2/siloxane composite containing 3 wt% SiO_2 was prepared as an example to show the heterogeneous degrowth. As-prepared, degrown, and regrown samples were fabricated as the same procedure shown in section 5.7. When we used fluorescence confocal microscopy to study the structure, we realized that the dye molecules were absorbed on the particles due to the electrostatic interaction between the basic imide moieties of PDI and the acidic species on the particle surface (Supplementary Figure 22), making the particles bright. To prove this mechanism, hydrophobic polytetrafluoroethylene (PTFE) particles that should prevent the absorption of the acidic species were used as the control sample. We first confirmed the absorption behavior of acid molecules by zeta potential measurement. It was found that the PTFE particles show near the same zeta potential as the intact one after treated by the method described in 4.3 (-84.2±6.7 mV for the PTFE particles and -83.6±10.6 mV for the acid-treated PTFE particles). In contrast, the same treatment changes the zeta potential of hydrophilic SiO_2 change from -6.16±0.69 mV to 6.79 ± 1.01 mV. These results indicated that no acid species were absorbed on the PTFE particle surface. We then studied the absorption of the dye molecules on the particle surfaces. Briefly, both SiO_2 and PTFE particles (0.3g) were first dispersed in the toluene (10g) by sonication, followed by the addition of the triflic acid and dye molecules in sequence or by the addition of only dye molecules. After 1h stirring, the particles were collected by centrifuged and the particles were rinsed three times with toluene. Supplementary Figure 23 shows the obtained particles. Only the hydrophilic SiO_2 obtained from the acid-present solution becomes light red. The fact the hydrophilic SiO_2 could not absorb dye molecules in the absence of acid species implied the significance of the acid in absorbing the dye molecules. PTFE particles are colorless and therefore no dye molecules were absorbed. Due to such labeling effect, we thus could evaluate matrix structure by the distribution of the bright particles. As shown in Figure 3fii, the particle concentrations at different z-positions of the as-prepared sample are nearly the same, indicating a homogeneous structure. In contrast, the degrown sample displays a particle concentration gradient from the top to the bottom (Figure 3fii in the main text), implying that degrowth occurred faster in the top region. After re-growth, a homogeneous distribution was

observed again, showing a structure-memory effect.

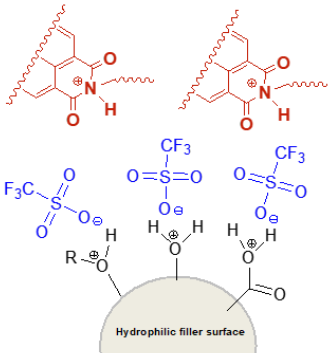

Supplementary Figure 22. Schematic illustration of the electrostatic interaction between the acid-absorbed particles and the PDI-based dye in SiO_2/siloxane composites.

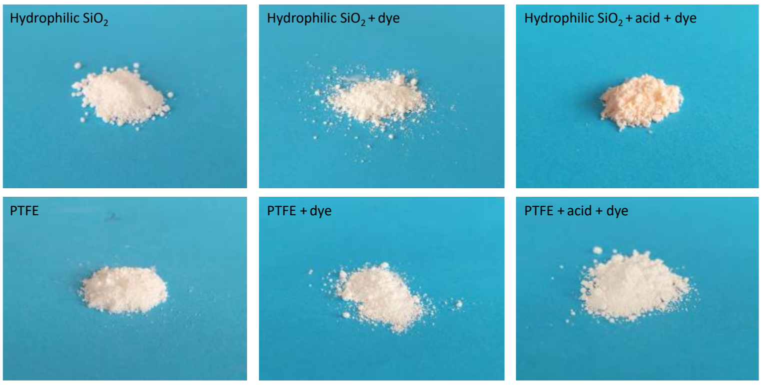

Supplementary Figure 23. Comparision of the dye-absorbing ability for the hydrophilic SiO_2 particles and hydrophobic PTFE particles in the absence/presence of triflic acid.

To further confirm the role of the immobilized active species for the heterogeneous degrowth, we also studied the bulk structure of the PTFE/siloxane composites in which the acid was not absorbed onto the PTFE particles. In this case, homogeneous degrowth was expected due to the absence of the immobilization effect. A dyed PTFE/siloxane composite was prepared and allowed to degrow and regrow as that described in Supplementary section 5.7. As expected, all the samples show homogenous matrices, i.e. the same fluorescence intensities at z-position (Supplementary Figure 24), indicating that particles themselves did not change degrowth manner.

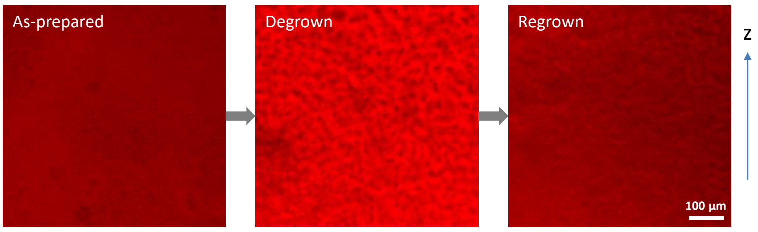

Supplementary Figure 24. Vertical cross-section fluorescence images of a dyed PTFE/siloxane composite at different states.

5.9 *Structure-memory effect during the heterogeneous degrowth-growth cycle*

As shown in Figure 3f, when the SiO_2-containing sample underwent a degrowth-growth cycle, a gradient structure formed at first in the degrowth process, which turned back into a homogeneous matrix again in the regrowth process. This phenomenon indicated that the region underwent faster degrowth (resulting in higher concentration of acid-adsorbed particles) would also undergo similarly faster growth. To confirm this mechanism, we prepared three kinds of degrown samples with different shrinking ratios, i.e. 10% degrown, 15% degrown and 20% degrown SiO_2/siloxane composites (the starting sample contained 3 wt% SiO_2), and compared their growth rates. Briefly, the degrown samples were immersed in a D_4 solution till they were swelled to 350 wt% of their initial weights, followed by storage in a sealed condition at 50 °C for different times. The samples were then washed by the hexane (containing 1% triethylamine) to remove residual monomer, followed by drying in air before weighted. The grown indices at different growth times were collected. As shown in Supplementary Figure 25, 10% degrown sample displays the slowest growth rate and the growth rate increases with the shrinking ratio, indicating that the higher particle concentrations in the degrown samples would lead to a faster growth rate. Therefore, the gradient structure in the degrown sample could regrow back to a homogenous structure.

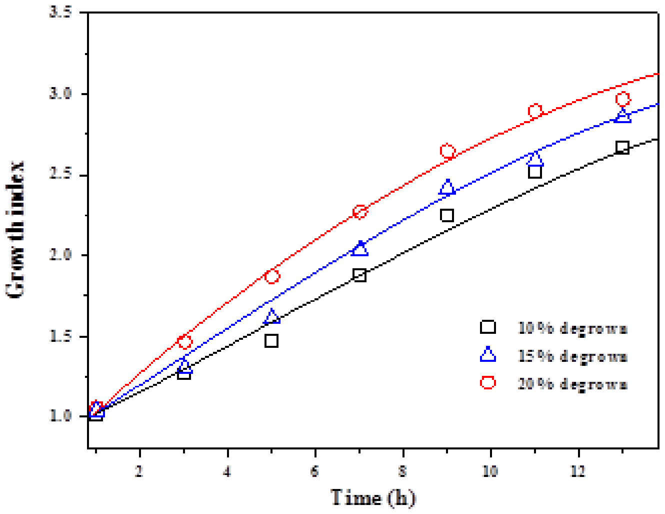

Supplementary Figure 25. Growth rates of the degrown SiO_2/siloxane composites with different shrinking ratios.

Supplementary Note 6: application demonstrations

6.1 *Growth demonstration*

Free growth: To prepare the samples shown in Figure 4a in the main text, the mixture solution of D_4, triD_4, and PDI-conjugated D_4 were cast on a silicon template with a square-hole array (height: 40 µm, length: 39 µm, wide: 39 µm), followed by addition of acid catalyst to trigger polymerization. After curing at rt overnight (12 h), the siloxane sample was peeled off and used as the starting substrate. The substrate was cut into several pieces which were then immersed in mixture solution with 6 wt% triD_4 for different times. After taken out from the solution, the samples were stored in sealed glass bottles for 4 days before further measurements. Normally,

three pieces were used as a group to test the repeatability.

Growth in H-shape chamber: To prepare the product shown in Figure 4b in the main text, an H-shape chamber consisting of a PTFE substrate with an H-shape groove and a PMMA cover with holes was used. The holes were designed for adding the D_4 and triD_4 mixture solution. As shown in Figure 4b in the main text, two dyed acidic living siloxane elastomers were placed in the two corners of the chamber, respectively. A mixture solution containing 6 wt% triD_4 was injected from one of the holes into the chamber. The solution containing 6 wt% triD_4 was used because the grown product obtained from it shows a modulus close to the one typically observed for the starting material. The holes were sealed, and the sample was stored at rt. During the polymerization, the conversion of monomer and crosslinker solution into solid materials (solid material has a density of 1.03 g/mL while the mixture solution has a density of 0.96 g/mL) reduced the volume of the system. Therefore, the mixture solution was replenished from time to time. During the growth, the chamber could be opened to check if the samples were in a solid state. After the mixture solution was injected, the samples swelled immediately and filled the corners in four hours. With further growth, the samples continued to expand along the walls and then also toward the vertical direction. After three weeks, the colored samples finally met in the center and joined together, resulting in an integrated H-shape elastomer. During this growth, the samples maintained a solid state. In the final product, the dye moieties in the samples have been redistributed throughout the sample (further proved by the fluorescence image), which indicated that the formation of the H-shape elastomer by a growing mechanism as proposed (but not by the polymerization on the surface of the samples).

6.2 *Self-healing tests*

In the presence of strong acid, the dynamic nature of siloxane networks should lead to excellent self-healing ability.[4,6] The self-healing ability is not only a useful property but also an indicator of the ongoing chain-exchange reactions and the presence of active living species in the samples. The self-healing behavior of the samples was estimated by both macroscopic observation and tensile tests. For macroscopic observation, the samples were cut into two pieces and then put together. After being stored in an inert atmosphere for 12 hours, the samples were then bent. The samples made from the deactivated samples were easily broken, while those made from active samples healed and exhibited similar flexibility as the intact uncut samples. The intact and healed samples were cut into typical "dog bone" shape and their mechanical properties were tested using a mechanical testing machine (Instron Model 5566). The loading rate was 20 mm/min. The sample size is 2.0 × 0.5 × 0.2 cm.

6.3 *Surface structure and property of the SiO_2/siloxane composites at different states*

A composite containing 3 wt% SiO_2 was allowed to undergo degrowth on a glass slide until an opaque surface was obtained (in air at 40 ºC for 96 h). The degrown sample was then regrown by feeding the D_4 monomer and a translucent coating was regained. SEM images of the as-prepared, degrown, and regrown samples showed that the as-prepared and the regrown samples have a flat surface, and the degrown sample have a rough surface (Supplementary Figure 26). The rough surface was composed of siloxane-coated SiO_2. The presence of silicone

was confirmed by EDX mapping in which element carbon was observed on the particles (Supplementary Figure 26). Therefore, the as-prepared sample was hydrophobic and the degrown sample was superhydrophobic.

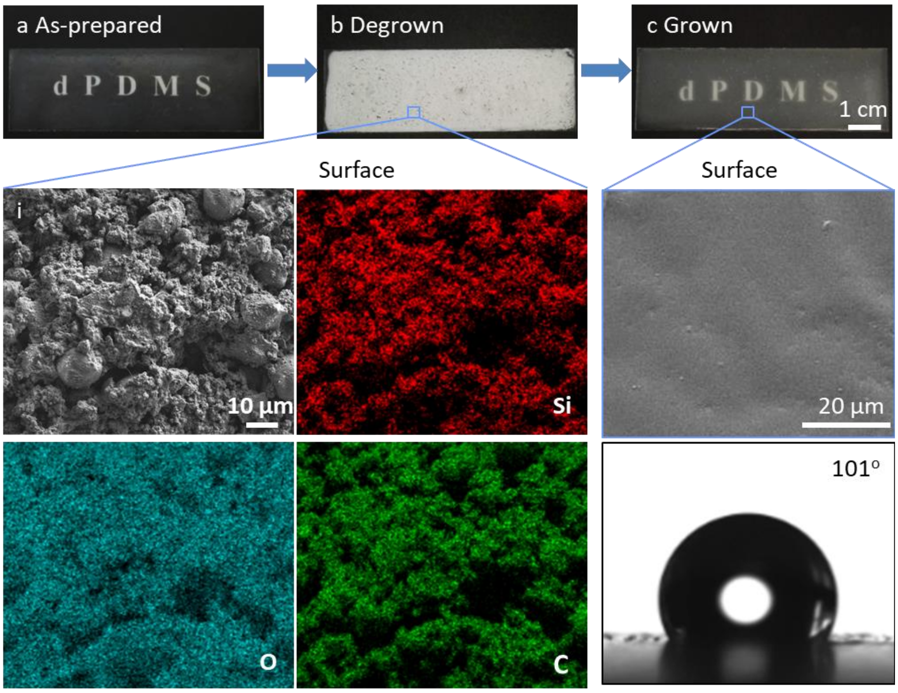

Supplementary Figure 26. Surface properties of the SiO_2/siloxane composites at different states. SEM was used to probe the surface morphology and EDX to analyze the elements. Water contact angle was employed to evaluate the surface property.

6.4 *Superhydrophobic living SiO_2/siloxane patterns*

3D profile of degrown surfaces: Both SiO_2/living siloxane and CB/living siloxane composites could be patterned by the described method. The SiO_2/siloxane samples containing 3wt% SiO_2 were used. The composite was coated on a glass slide, followed by covering Teflon models on the samples (Figure 4d in the main text). The patterns were obtained after degrowing in air at 40 °C for 96 h. For erasing the patterns on the surface, the patterned sample was fed with D_4 monomer to allow it to grow back to its initial weight. Two masks (Supplementary Figure 27a-b) were used to show the rewritability. Supplementary Figure 27c-e shows the 3D profile of the stripe pattern. The uncovered regions shrunk and showed a lower horizontal line than the covered regions.

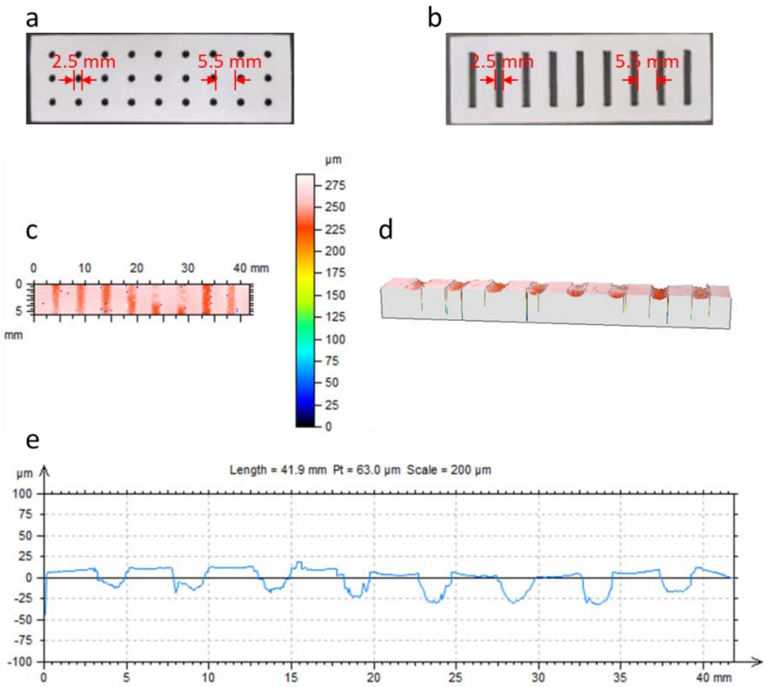

Supplementary Figure 27. Profiles of the patterned surfaces. a&b) Masks used for making the patterns, c) 2D topography, d) 3D topography, e) Line roughness.

Stability: Patterning SiO_2/siloxane composite film was used to evaluate the stability of deactivated samples. The sample was deactivated by triethylamine, as described in Methods section and then annealed at 70 ^{o}C in a sealed condition for 24h. As shown in Supplementary Figure 28, the pattern was stable.

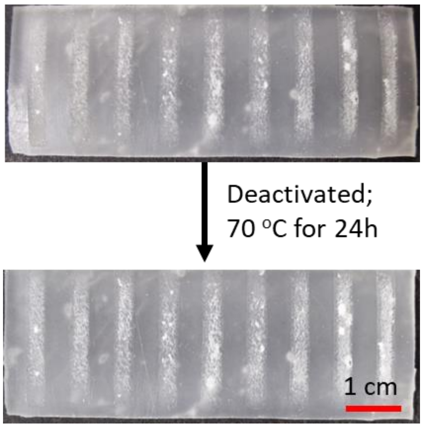

Supplementary Figure 28. Photograph of the deactivated patterning SiO_2/siloxane composite film after being treated at 70 ^{o}C for 24h.

.

6.5 *Reversible actuation during the degrowth-growth cycle*

The sample shown in Figure 4e in the main text was prepared by tailoring from a film. Each arm is about 3.2(l)$\times$0.6(w)$\times$0.2(t) cm. The sample was then put on a substrate with one

surface exposed to the air and one surface contact with the substrate. It was assumed that no evaporation would occur on the surface contacted with the substrate and the small molecules escaped from one side (the surface exposed to the air) only. The arms bent toward the evaporated side gradually. For regrowth, D_4 monomer solution was dropped to the degrown sample (the liquid was absorbed soon, rather than concentrated on the surface), followed by storage in a sealed plastic bottle for growth. The bent sample turned flat slowly in 3 days.

To demonstrate the actuating effect of the degrowth, a CB/siloxane composite rope was prepared by curing the composite in a plastic straw. We used the obtained rope to tie a toy car to a substrate and allowed the sample to degrow in air at rt. As shown in Supplementary Figure 29a, after degrowth for 120 h, the rope has shrunk and stretched the car close to the pinning point. On the other hand, we also used the ropes to tie the paper-art (Supplementary Figure 29b). After having been allowed to degrow in air at rt for 96 h, the ropes also shrunk to open the paper art. We then provided a D_4 monomer solution to the rope by dropping the monomer solution to the rope to trigger growth. The paper art was closed again.

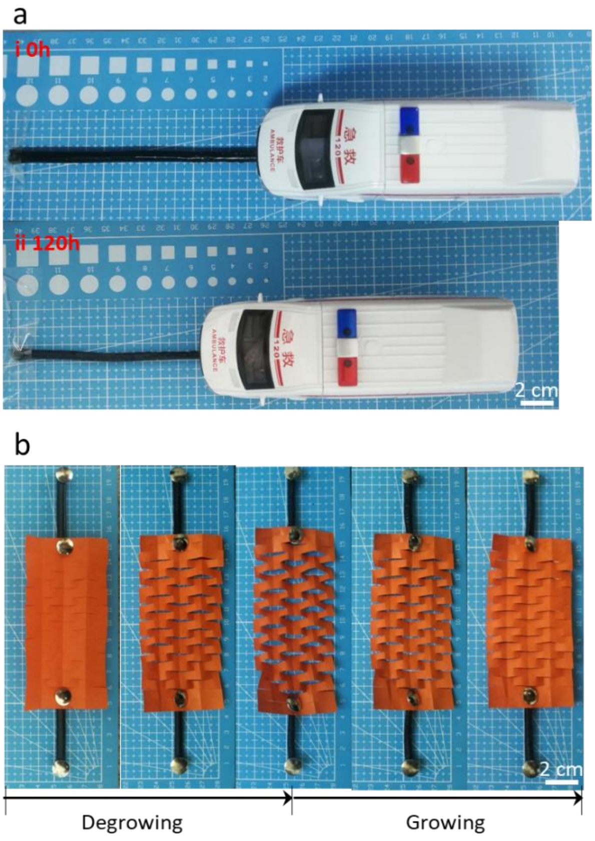

Supplementary Figure 29. Actuation effect of CB/siloxane composites. a) Automatically drawing a car during degrowth, b) Reversible paper art opening/closing in a degrowth-growth cycle.

Supplementary References

1 Chojnowski, J. Kinetically controlled siloxane ring-opening polymerization. *J. Inorg. Organomet. Polym.* **1**, 299-323 (1991).

2 Kantor, S. W., Grubb, W. T. & Osthoff, R. C. The mechanism of the acid-and base-catalyzed equilibration of siloxanes. *J. Am. Chem. Soc.* **76**, 5190-5197 (1954).

3 Hurd, D. T. On the mechanism of the acid-catalyzed rearrangement of siloxane linkages in organopolysiloxanes. *J. Am. Chem. Soc.* **77**, 2998-3001 (1955).

4 Chang, P. S. & Buese, M. A. Silicone networks prepared via a living percolation mechanism: postgelation structure for networks with a variety of junction functionalities. *J. Am. Chem. Soc.* **115**, 11475-11484 (1993).

5 Bennardi, D. O., Romanelli, G. P., Autino, J. C. & Pizzio, L. R. Trifluoromethanesulfonic acid supported on carbon used as catalysts in the synthesis of flavones and chromones. *Catal. Commun.* **10**, 576-581 (2009).

6 Zhou, X., Zhang, X., Zhao, H., Krishnan, B. P. & Cui, J. Self-Healable and Recyclable Tactile Force Sensors with Post-Tunable Sensitivity. *Adv. Funct. Mater.* **30**, 2003533 (2020).

7 Di Noto, V., Negro, E., Sanchez, J.-Y. & Iojoiu, C. Structure-relaxation interplay of a new nanostructured membrane based on tetraethylammonium trifluoromethanesulfonate ionic liquid and neutralized nafion 117 for high-temperature fuel cells. *J. Am. Chem. Soc.* **132**, 2183-2195 (2010).

8 Vernerey, F. J., Long, R. & Brighenti, R. A statistically-based continuum theory for polymers with transient networks. *J. Mech. Phys. Solids* **107**, 1-20 (2017).

9 Routh, A. F. Drying of thin colloidal films. *Rep. Prog. Phys.* **76**, 046603 (2013).

10 Karadağ, E., Üzüm, Ö. B. & Saraydin, D. Swelling equilibria and dye adsorption studies of chemically crosslinked superabsorbent acrylamide/maleic acid hydrogels. *Eur. Polym. J.* **38**, 2133-2141 (2002).

11 Yiamsawas, D., Kangwansupamonkon, W., Chailapakul, O. & Kiatkamjornwong, S. Synthesis and swelling properties of poly [acrylamide-co-(crotonic acid)] superabsorbents. *React. Funct. Polym.* **67**, 865-882 (2007).
